# A Real-World Investigation of MRI Changes in Bone in Patients with Type 1 Gaucher Disease Treated with Velaglucerase Alfa: The EIROS Study

**DOI:** 10.3390/jcm13102926

**Published:** 2024-05-16

**Authors:** Monia Bengherbia, Marc Berger, Bénédicte Hivert, Florian Rigaudier, Luc Bracoud, Ole Vaeterlein, Karima Yousfi, Michele Maric, Marie Malcles, Nadia Belmatoug

**Affiliations:** 1 Department of Internal Medicine, Referral Center for Lysosomal Diseases, Beaujon Hospital, AP-HP, Université Paris Cité, 92110 Clichy, France; monia.belhaoua@takeda.com (M.B.); karima.yousfi@aphp.fr (K.Y.); 2 Department of Biological and Clinical Hematology, Estaing Hospital, CHU Clermont-Ferrand, 63000 Clermont-Ferrand, France; mberger@chu-clermontferrand.fr; 3 Department of Hematology, Saint Vincent de Paul Hospital, GHICL, 59000 Lille, France; hivert.benedicte@ghicl.net; 4 CEN Biotech, 21000 Dijon, France; florian.rigaudier@groupecen.com; 5 Clario Inc. (Formerly Bioclinica, Inc.), 69006 Lyon, France; luc.bracoud@clario.com; 6 Clario Inc. (Formerly Bioclinica, Inc.), 20355 Hamburg, Germany; ole.vaeterlein@clario.com; 7 Takeda France SAS, 75116 Paris, France; michele.maric@takeda.com (M.M.); marie.malcles@takeda.com (M.M.)

**Keywords:** Gaucher disease, lysosomal storage disorder, bone marrow infiltration, hepatosplenomegaly, thrombocytopenia, real-world data, enzyme replacement therapy, velaglucerase alfa, magnetic resonance imaging

## Abstract

**Background/Objectives**: Gaucher disease type 1 (GD1) is characterized by hepatosplenomegaly, thrombocytopenia, and disabling bone manifestations requiring regular MRI monitoring. The EIROS study assessed the real-world impact of velaglucerase alfa on GD1 bone disease, using MRI data collected in French clinical practice. **Methods**: MRIs collected retrospectively from treatment initiation and prospectively during follow-up (12-months) were analyzed centrally by a blinded expert radiologist to evaluate bone infiltration using the Bone Marrow Burden (BMB) score and a qualitative method (stable, improved or worsened for the spine and femur). Abdominal MRIs were also centrally analyzed to assess hepatosplenomegaly. Bone manifestations, hepatosplenomegaly, and hematologic parameters were analyzed from medical records. **Results**: MRI data were available for 20 patients: 6 treatment-naive patients and 14 patients who switched to velaglucerase alfa from another GD treatment. Interpretable MRIs for BMB scoring were available for seven patients for the spine and one patient for the femur. Qualitative assessments (*n* = 18) revealed stability in spine and femur infiltration in 100.0% and 84.6% of treatment-switched patients (*n* = 13), respectively, and improvements in 80.0% and 60.0% of treatment-naive patients (*n* = 5), respectively; no worsening of bone infiltration was observed. Liver, spleen, and hematologic parameters improved in treatment-naive patients and remained stable in treatment-switched patients. **Conclusions**: The qualitative real-world data support findings from clinical trials suggesting the long-term effectiveness of velaglucerase alfa on GD1 bone manifestations. When MRI assessment by radiologists with experience of GD is not possible, a simplified qualitative assessment may be sufficient in clinical practice for monitoring bone disease progression and treatment response.

## 1. Introduction

Gaucher disease (GD) is a rare, autosomal recessive, metabolic disorder caused by pathological variants in the *GBA1* gene leading to deficiency in the activity of the lysosomal enzyme glucocerebrosidase. The disease is characterized by the progressive accumulation of glucosylceramide and glucosylsphingosine in the lysosomes of macrophages (named Gaucher cells)—particularly in the liver, spleen, and bone marrow—resulting in multisystemic disease and heterogenous clinical manifestations. Three main clinical forms of GD have been described [1,2]. GD Type 1 (GD1; OMIM: 230800, ORPHA: 77259) is characterized by hepatosplenomegaly, thrombocytopenia and bone involvement, with the severity and age of onset of disease ranging from severe during childhood to patients who remain asymptomatic throughout life. Type 2 (GD2; OMIM230900, ORPHA: 77260) is an acute neuronopathic disease with hepatosplenomegaly and very early onset with death during infancy. Type 3 (GD3; OMIM 231000, ORPHA: 77261) is a chronic neuronopathic form associated with onset in childhood or adolescence, and systemic symptoms similar to those observed in GD1 [1,2].

The estimated prevalence of GD has been reported to range from 1 to 2 per 100,000 in the general population, but is much higher (118 in 100,000) in the Ashkenazi Jewish population [2]. GD1 is the most common form, accounting for around 94% of GD cases in Western countries [2]. As a chronic illness, GD1 can lead to significant morbidity and can have a major impact on patient quality of life. In particular, the highly prevalent bone manifestations in GD1—which include bone marrow infiltration, Erlenmeyer flask-shaped deformation, bone infarcts, avascular osteonecrosis, osteopenia, osteoporosis, osteolytic lesions, fractures, secondary osteoarthritis, and osteosyntheses—are the main causes of pain and disability [3].

There are currently two therapeutic strategies for the treatment of GD: enzyme replacement therapy (ERT) and substrate reduction therapy (SRT). ERT is widely used as the first-line treatment and two intravenously administered ERTs have received marketing authorization in the EU, imiglucerase (Cerezyme^®^) [4] and velaglucerase alfa (VPRIV^®^) [5]. Both substances have been shown to be safe and effective, leading to improvements in the hematologic and visceral manifestations of GD [6,7,8,9,10,11]. Although ERT cannot reverse pre-existing permanent bone damage, data from clinical trials have indicated that long-term ERT is associated with a reduction in bone crises and bone pain, an improvement in bone mineral density, and a decrease in bone marrow infiltration based on bone marrow burden (BMB) scores [11,12,13,14,15,16].

Various recommendations for the overall management of patients with GD being treated with ERT have been published based on evaluations by international and national working groups [17,18,19,20,21,22,23], but there has been little consensus when it comes to defining treatment algorithms and monitoring protocols. However, management protocols have been proposed by national healthcare providers, and clear short-term and long-term treatment goals have been defined [24,25,26]. In addition, analyses of data collected by national [27,28] and international registries [29,30,31,32] have provided valuable insights into disease outcomes and improved understanding of the natural history of GD. Despite these advances, the real-world impact of ERT on the bone pathophysiology associated with GD remains poorly understood, prompting the publication of detailed expert recommendations for the monitoring of bone alterations with the aim of improving the management and follow-up of bone disease [3,33]. These practical recommendations highlighted the complexity of bone involvement in GD and the need for radiologists experienced in GD to be included in the multidisciplinary management team. The key recommendations for evaluating bone involvement included the use of MRI as the gold standard for assessing bone marrow infiltration in the lumbar spine and lower extremities, with semiquantitative analyses using either the Düsseldorf Gaucher or BMB scores reserved for clinical studies involving adults, and for monitoring avascular osteonecrosis and bone infarcts. X-rays were recommended for identifying specific lesions, cortical thinning, lytic lesions, fractures, osteosyntheses, and osteoarthritis. Standardized regular dual-energy X-ray absorptiometry (DXA) assessments of bone mineral density were recommended to evaluate osteopenia and osteoporosis [3].

In France, patients with GD are managed according to a National Protocol for Diagnosis and Care (PNDS [26]) by a network of specialized referral centers for lysosomal diseases, and data are collected in a national GD registry [27], all of which is facilitated by a multidisciplinary committee for the evaluation and treatment of GD (Comité d’Évaluation du Traitement de la maladie de Gaucher; CETG) [34]. The PNDS contains guidelines for the monitoring of bone involvement and refers to detailed procedures developed for radiologists carrying out abdominal and bone MRIs in patients with GD [35].

This current real-world observational study (EIROS) was carried out to gain a better understanding of the evolution and outcomes of bone disease in patients with GD1 being treated with velaglucerase alfa, and was conducted in response to the ongoing evaluation by the French national health authority (Haute Autorité de Santé; HAS) and to complement the data collected by the Gaucher Outcome Survey (GOS) registry [29]. Thus, the main objective of this study was to evaluate the progression of bone disease in these patients using MRI data obtained by individual radiologists in real-world practice. Given the complexity of bone imaging in patients with GD and the high level of expertise required to interpret the data, the MRIs were transferred to a specialized center and a standardized evaluation and analysis by an expert radiologist was conducted for the purpose of the study. Other objectives were the evaluation of changes in other key disease characteristics including organomegaly, clinical manifestations (acute and chronic bone pain), and biological parameters.

## 2. Materials and Methods

### 2.1. Study Design and Patients

This observational study of patients with GD1 treated with velaglucerase alfa was conducted using data collected retrospectively and prospectively in everyday clinical practice. The study was conducted between March 2017 and August 2019. French hospital centers involved in the management of more than one patient with GD1 being treated with velaglucerase alfa were invited to participate in the study. Participating centers were asked to provide data collected from patient medical records for all patients with a confirmed diagnosis of GD1 who were being treated with velaglucerase alfa and who had available digital MRI data from an examination conducted within the 5 years preceding velaglucerase alfa initiation, or within the 3 months following treatment initiation. The MRIs could have been conducted in either a hospital radiology unit or local radiology center. Prior to starting velaglucerase alfa treatment, patients could either be treatment-naive or have been treated previously with any other GD treatment. Patients participating in an ongoing blinded clinical trial were excluded. All patients, or their parents or legal representative, provided written informed consent before being included in the study.

### 2.2. Data Collection

**Figure 1:**
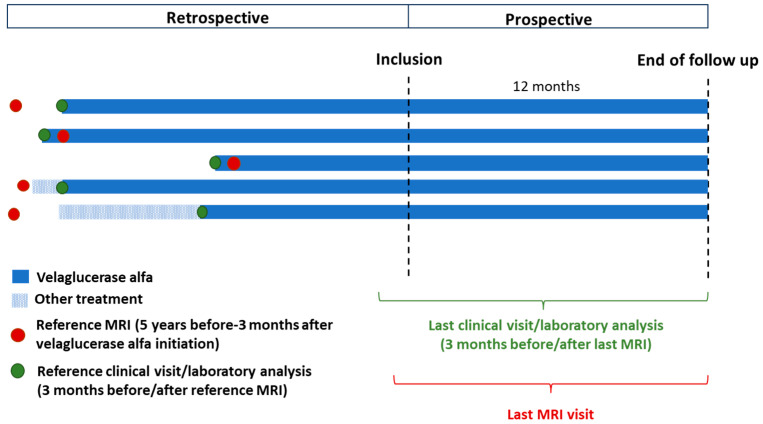
Summary of the study design. The reference MRI was determined individually for each patient and was the MRI performed closest to the time of velaglucerase alfa initiation (conducted in the five years preceding the start of velaglucerase alpha treatment or in the three months following velaglucerase alfa initiation). In France, MRIs are recommended to be performed at treatment initiation, 1 year after initiation and then every 2 years; thus, the last MRI may have been performed in the year before inclusion or during the 12-month follow-up period. Clinical and biological data were collected for examinations performed either three months before or three months after each MRI, and at velaglucerase alfa initiation.

All data analyzed in this study were collected from patient medical records on routine imaging, clinical examinations and biological analyses that were part of the standard medical care and systematic follow-up of patients with GD1 by the physicians responsible for their management. A summary of the study design and data collected is shown in Figure 1.

For the retrospective analysis, data were analyzed for each patient from the date of the MRI closest to the initiation of velaglucerase alfa (reference MRI) to the date of inclusion in the study. For inclusion in the study, the reference MRI had to be conducted within the 5 years preceding velaglucerase alfa initiation, or within the 3 months following treatment initiation. In the prospective analysis, data were analyzed from all visits from inclusion until the date of the last MRI before the end of the study follow-up period (12 months after inclusion).

### 2.3. Analysis of Imaging Data

All available digital MRI data (bone MRIs and abdominal MRIs) were collected, anonymized, transferred to a central imaging lab (Clario, formerly known as Bioclinica), and then analyzed by a single radiologist with experience in GD. This central radiologist was blinded to all clinical and demographic data but had access to data from all available MRI visits and sequences, in chronological order of acquisition. The bone MRIs used to determine BMB scores (as described previously [36] by Clario, Hamburg, Germany). For the abdominal MRIs, liver and spleen volumes were semi-automatically measured by trained and experienced MRI technicians (Clario, Lyon, France), based on axial T1-weighted and T2-weighted sequences, respectively, or on the most suitable available MRI sequence if these were missing or of poor quality. Boundaries of both organs were carefully reviewed on each MRI slice, when visible, and the corresponding volumes were automatically derived in mm^3^ based on image resolution, and then expressed as multiples of normal (MN) based on 25 mL/kg of body weight for the liver and 2 mL/kg of body weight for the spleen, as described previously [25].

Reports of imaging investigations (abdominal MRI for monitoring of hepatosplenomegaly and standard X-rays or MRI for bone lesions) written by local radiologists at each visit were collected from medical records.

### 2.4. Analysis of Demographic, Clinical, and Biological Data

Data concerning patient demographics and disease, treatment history and genotypes were collected at inclusion. Data were also collected on the dose, frequency, and duration of velaglucerase alfa treatment, the use of concomitant treatments, and the occurrence of any adverse events during the study period. The time between visits was also recorded for each variable.

Both retrospective and prospective clinical and biological data were analyzed from patient medical records of examinations and analyses performed within three months of each MRI and at velaglucerase alfa initiation (Figure 1). The clinical data included body mass index (BMI), and the occurrence of acute and chronic bone pain, and clinical assessments of hepatomegaly and splenomegaly. For the biological analyses, all available data were collected and data concerning hemoglobin concentrations, platelet counts and chitotriosidase activity were analyzed relative to published reference standards. For hemoglobin, normal levels were defined as ≥12.0 g/dL for men and ≥11.0 g/dL for women and children (≤12 years of age) [25,29]. Thrombocytopenia was classified as normal or mild (not clinically significant) for platelet counts > 120.0 × 10^9^/L; moderate for counts < 120.0 × 10^9^/L to ≥60 × 10^9^/L; and severe for counts < 60 × 10^9^/L [25,29]. The upper limit for chitotriosidase activity, an established marker for GD [37], was compared to the median levels reported in individuals without GD (median [range]: 20 [4–76] nmol/mL/h) [38].

### 2.5. Study Endpoints

The primary endpoint was the change in BMB score between the reference MRI and the last follow-up MRI determined by the expert-radiologist centralized analysis. The secondary endpoints included the change between the reference visit and the last follow-up examination for the following variables: the presence of bone infiltration according to imaging reports written by local radiologists; the occurrence of hepatosplenomegaly and of acute and chronic bone pain according to clinical records; spleen and liver volumes determined by the expert-radiologist centralized analysis of abdominal MRIs; and biological parameters according to medical records. Bone lesions, bone pain, treatment duration, changes in liver and spleen volumes and biological parameters, as well as the slope of these changes, were also assessed according to treatment history (i.e., in treatment-naive patients compared to those switching to velaglucerase alfa from another GD treatment).

### 2.6. Statistical Methods

Descriptive data were described as means (± standard deviation) for quantitative variables and as numbers and percentages (*n* %) for qualitative variables. After analysis of the distribution of all variables, changes in BMB scores and in MRI liver and spleen volumes between MRIs were analyzed using the Wilcoxon rank test. Between-visit changes in clinical characteristics and MRI bone characteristics were analyzed using the McNemar chi-square test. For biological analyses, between-visit changes and differences in values between treatment-naive and treatment-switched patients were analyzed using the *t* test or the Wilcoxon rank test. Missing data were treated as such, and no imputation of missing values was performed. Only variables for which data were available for more than 25% of the patients were included in the analysis. *p* values < 0.05 were considered significant. All statistical analyses were carried out using SAS^®^ software (version 9.4).

### 2.7. Post Hoc Analysis

A centralized post hoc analysis was performed by the expert radiologist to qualitatively assess the change in bone infiltration from the first to last available MRIs. Changes were rated as stable, improved, or worsened and were scored for the spine and femur separately. The presence of emerging new spine and femur events was also documented. The changes were analyzed for the whole population and according to treatment history (treatment-naive patients versus treatment-switched patients), time between the first and last MRI, and duration of treatment (velaglucerase alfa alone and velaglucerase alfa plus any prior treatment). Differences in the proportion of treatment-naive patients and treatment-switched patients with improved, stable, or worsened characteristics were analyzed using the Fisher test, and all other post hoc analyses were analyzed using descriptive statistics.

## 3. Results

### 3.1. Study Population

**Figure 2:**
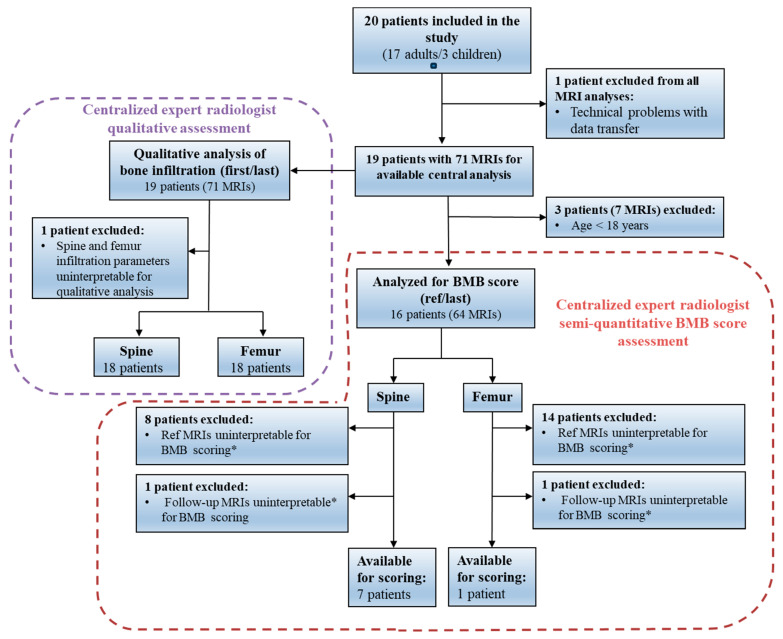
Patient flow through the study for the centralized semi-quantitative BMB score assessment of bone infiltration (red box) and the post hoc centralized qualitative assessment of bone infiltration (purple box). Abbreviations: BMB score, bone marrow burden score. * The main reasons for MRIs being uninterpretable for BMB scoring were the use of incorrect scanning parameters (acquisition of T1- and T2-weighted sequences with fat suppression) and/or incomplete imaging of the required anatomical region (particularly the absence or incomplete imaging of the distal femur).

A total of 20 patients (17 adults and 3 children) managed at nine hospital centers were included in the study between 1 March 2017 and 27 December 2018. The first retrospective MRI took place on 17 March 2006, and the last prospective follow-up visit took place on 16 August 2019. One patient was excluded from the MRI analyses due to technical problems with the storage and transfer of the MRI data (Figure 2).

### 3.2. Demographic and Clinical Characteristics

The demographic, bone imaging, and clinical characteristics of the patients are shown in Table 1. The mean age of the adult patients at inclusion was 52 ± 12 years (range 28–75 years) and the ages of the three children at inclusion were 8, 10 and 15 years. Three patients had undergone splenectomy before the start of the study. Biological data and the results of genotyping analyses are also listed in Table 1.

### 3.3. Treatment Patterns and Exposure

The mean duration of velaglucerase alfa treatment, from initiation to the last study follow-up visit, was 5.2 ± 2.6 years (*n* = 19). Six of the patients included in the study (three children and three adults) were naive to GD treatment before velaglucerase alfa initiation. The mean time between GD1 diagnosis and treatment initiation for these patients was 2.6 ± 4.3 years and the mean duration of treatment with velaglucerase alfa was 2.6 ± 1.4 years. Among the fourteen patients who switched to velaglucerase alfa, 93% switched from another ERT (imiglucerase) and one patient switched from an SRT (miglustat). The mean time between GD1 diagnosis and initiation of treatment with any type of GD therapy for these patients was 9.9 ± 10.9 years and the mean duration of velaglucerase alfa treatment was 6.4 ± 2.1 years.

The mean dose of velaglucerase alfa administered during the study was 56.8 ± 12.7 U/kg (*n* = 19). Analysis of dose frequency showed that most patients (*n* = 14) received the treatment fortnightly (every 14 or 15 days), as recommended [5], whereas the remaining patients received treatment every 17 days (*n* = 1), every 21 days (*n* = 3) or monthly (*n* = 1). Minor modifications to the dose and/or frequency of velaglucerase alfa administration were made for nine patients. One patient temporarily stopped treatment with velaglucerase alfa (for 112 days), and then later switched to an oral treatment (eliglustat). The total duration of velaglucerase alfa treatment in this patient was 6.3 years. No adverse events were reported during the study period.

Overall, 13 patients (65%) were prescribed a concomitant treatment during the study period, including analgesics (6/20), vitamin D supplements (7/20) and specific treatments for osteoporosis (2/20).

### 3.4. Evolution of Bone Marrow Burden Scores according to the Centralized Analysis

A total of 71 digital MRI records (58 collected retrospectively and 13 collected prospectively), with an average of around 4 MRIs per patient (range: 2–7), were available for centralized analysis. However, the number of optimal or interpretable MRIs for assessment of the BMB score was limited (Figure 2). Seven of the MRIs (five collected retrospectively and two collected prospectively) were excluded from the BMB analysis because they were from examinations conducted on children, for whom calculation of the BMB score has previously been found to be unreliable [3]. Out of the remaining 64 MRIs analyzed, 23 (35.9%) were deemed uninterpretable for evaluation of the BMB spine score and 55 (85.9%) were deemed uninterpretable for evaluation of the femur BMB score. The most common causes for the insufficient quality of the MRIs were the use of incorrect scanning parameters (acquisition of T1- and T2-weighted sequences with fat suppression preventing proper comparison of MRI signal intensity with that of subcutaneous fat [femur] and non-diseased intervertebral discs [spine], as originally required for BMB scoring) and/or incomplete imaging of the required anatomical region (particularly the absence or incomplete imaging of the distal femur). In total, only seven patients had interpretable data for BMB spine scoring from both the reference MRI (mean score: 3.6 ± 2.1) and a follow-up MRI (mean score: 2.9 ± 1.1). The average time between the reference MRI visit and last MRI visit for calculation of the BMB score was 4.3 years ± 2.9 years. No significant change in the mean BMB spine score was observed between these reference and last MRIs (Δ −0.7 ± 1.5; *p* = 0.25). Only one patient had interpretable data for BMB femur scoring from both the reference MRI and a follow-up visit. As a result, no calculation could be performed for the change in femur BMB score or total BMB score for the whole population. As an alternative, a centralized post hoc qualitative assessment of the change in bone infiltration in the spine and femur from the first to last MRI data was performed by the expert radiologist.

### 3.5. Centralized Post Hoc Qualitative Analysis of the Evolution of Bone Infiltration in the Spine and Femur

**Figure 3:**
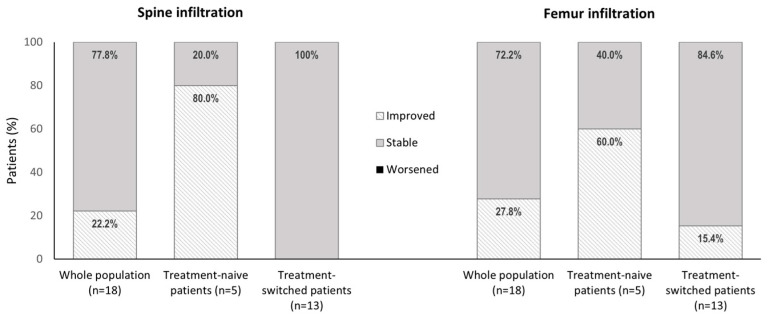
Evaluation of the qualitative change in spine and femur infiltration between the first and last available MRIs for the whole population (*n* = 18) and according to treatment patterns. MRI data were uninterpretable for this centralized expert-radiologist qualitative analysis for one of the treatment-naive patients. Mean times between the first and last MRIs were 4.9 years ± 2.6 for the whole population (*n* = 18), 3.6 ± 1.4 for treatment-naive patients (*n* = 5), and 5.5 ± 2.9 for patients who switched to velaglucerase alfa from another Gaucher disease treatment (*n* = 13). No patients showed worsening of spine or femur infiltration.

Overall, 18 patients (5 treatment-naive patients and 13 treatment-switched patients) had interpretable MRIs for the centralized expert-radiologist post hoc qualitative evaluation of the change in spine or femur infiltration (Figure 2). Improvements in spine and femur infiltration were observed for 22.2% (*n* = 4/18) and 27.8% (*n* = 5/18) of patients, respectively, and stability was observed for 77.8% (*n* = 14/18) and 72.2% (*n* = 13/18) of patients, respectively. None of the patients had worsening spine or femur infiltration between the first and last MRIs (Figure 3).

Significant differences in the proportion of patients who had improved or stable spine and femur infiltration between the first and last MRIs were observed for treatment-naive patients compared to treatment-switched patients (*p* < 0.001 for the spine and *p* < 0.05 for the femur). The proportion of patients with an improvement in infiltration was highest among treatment-naive patients: improvements in spine infiltration were observed for 80.0% (*n* = 4/5) of these patients and improvements in femur infiltration were observed for 60.0% (*n* = 3/5). In contrast, spine infiltration remained stable for all patients (*n* = 13) who had switched to velaglucerase alfa from another GD treatment. An improvement in femur infiltration was observed for two of the treatment-switched patients (15.4%), with femur characteristics in the eleven remaining patients (84.6%) remaining stable between MRIs (Figure 3; *p* values for between-group differences in the proportion of patients in each class < 0.05).

The change in spine and femur infiltration also varied depending on the duration of velaglucerase alfa treatment and of velaglucerase alfa and any prior treatments (Table 2). The mean duration of treatment was shorter for patients who had an improvement in spine infiltration (3.5 ± 0.3 years both for velaglucerase alfa alone and for velaglucerase alfa and prior treatment) than for patients with stable spine infiltration (6.0 ± 2.5 years for velaglucerase alfa alone and 17.0 ± 7.2 years for velaglucerase alfa and prior treatment). The same trend was observed for the change in femur characteristics: mean treatment duration for patients with an improvement in femur characteristics was 4.7 ± 1.7 for velaglucerase alfa alone and 10.1 ± 9.2 for velaglucerase alfa and prior treatment, compared to 5.7 ± 2.6 for velaglucerase alfa alone and 15.5 ± 8.2 for velaglucerase alfa and prior treatment for patients with stable femur characteristics (Table 2).

### 3.6. Review of Bone Imaging Data and Clinical Bone Manifestations according to Medical Records

The collection of bone imaging reports written by local radiologists indicated that 17 of the 19 patients had bone lesions assessed by standard X-Rays and MRI at the reference visit and at the last visit (Table 3). One new case of bone infiltration (in a treatment-naive patient) was reported by local radiologists at the last visit compared to the reference visit. This new case of bone infiltration occurred in the patient for whom the data were deemed uninterpretable for the post hoc centralized expert-radiologist qualitative analysis. One case of bone infiltration present at the reference MRI was reported as absent at the last MRI. However, bone infiltration in this patient (a treatment-naive patient aged 15 years) was noted as present at the last visit by the central radiologist. Other bone lesions deemed unlikely to be related to GD were reported in 12 patients (63%), with the most common lesions including vertebral hemangiomas and degenerative disc disease (each present in two patients: 10.5% of the study population).

In the clinical examinations, no statistically significant changes in the overall number of patients with acute or chronic bone pain were reported (Table 3). No new occurrences of acute bone pain were reported during the study and neither of the two patients with mild (*n* = 1) or moderate (*n* = 1) acute bone pain at the reference visit reported having acute bone pain at the last visit. Data on the change in severity of chronic bone pain were available for six patients, all of whom had mild or moderate pain at the reference visit. Four of these patients had no chronic bone pain at the last visit, and the mild (*n* = 1) and moderate (*n* = 1) pain in the remaining patients remained stable.

### 3.7. Evolution of Liver and Spleen Parameters

The number of patients in the whole population with hepatosplenomegaly, evaluated by MRI and/or by clinical examination, did not change significantly over the course of the study (Table 4).

A total of 38 abdominal MRIs were conducted during the study period and all these MRIs contained interpretable data: 33 (86.8%) and 31 (81.6%) of the MRIs were deemed optimal for liver and spleen volume measurements, respectively, whereas 7 (18.4%) and 5 (13.2%) of the MRIs were deemed suboptimal but interpretable, respectively, mainly due to motion artifacts or suboptimal anatomical coverage. As abdominal MRIs had only been conducted for a limited number of patients at both a reference visit and follow-up visit (liver: 7 patients; spleen; 6 patients), the centralized analysis of the evolution of spleen and liver volume was evaluated from the first available MRI to the last MRI. The mean time between the first and last visits was 5.6 ± 2.9 years for the liver analysis and 5.6 ± 3.1 years for the spleen analysis (compared to mean times of 5.6 years ± 3.7 for liver analysis and 5.4 years ± 4.0 for spleen analysis between the reference and last MRIs). The mean estimated liver and spleen volumes at the first and last MRIs for the whole population and according to treatment history are shown in Table 4.

The mean liver volume in patients who had switched from a prior treatment (*n* = 9) was 0.9 ± 0.2 MN at the reference MRI and remained stable at the last MRI (mean: 0.8 ± 0.1 MN; Δ + 61.7 ± 79.7 mL). Similarly, spleen volumes remained largely stable in the treatment-switched patients: 3.2 ± 1.4 MN at the reference MRI and 2.6 ± 1.0 MN at the last visit (Δ − 86.9 ± 114.6 mL).

In the treatment-naive patients (*n* = 3), liver volumes were on average 1.8 ± 1.1 MN at the reference MRI, but decreased by −562.6 ± 777.0 mL to 1.2 ± 0.23 MN at the last visit. Spleen volumes in these patients were considerably above normal at the reference MRI (mean: 16.3 ± 19.8 MN) but decreased by the last MRI (Δ − 1099.6 ± 1497.4 mL) to 3.9 ± 2.8 MN.

### 3.8. Evolution of Biological Parameters

The mean changes in hemoglobin concentrations, and in platelet counts and chitotriosidase activity for the whole population between the first and last analyses are shown in Table 5.

The hemoglobin concentration in patients that had switched from a prior treatment was in the normal range (≥11.0–12.0 g/dL, depending on age and gender) for all patients at the reference analysis, and remained stable with no significant change being observed between analyses (mean: 14.3 g/dL at both visits; slope: −0.1 g/dL/year; Table 5). In contrast, the mean hemoglobin concentration in treatment-naive patients was slightly below normal at the reference visit (mean: 10.8 g/dL), but increased to within the normal range for all patients at the last visit (mean: 12.8 g/dL; slope: 11 g/L per year; Table 5).

Among the treatment-switched patients (*n* = 12), platelet counts at the reference analysis were within the normal/mild range (≥120 × 10^9^/L) for eight patients, but moderate thrombocytopenia (platelet counts ≤ 120 × 10^9^/L but ≥60 × 10^9^/L) was present in four patients. Platelet counts increased or remained stable at the last analysis for all the patients who switched treatments (mean: 169.9 ± 35.2 × 10^9^/L; Δ: +23.2 ± 27.3 × 10^9^/L), although the moderate thrombocytopenia persisted in one patient. For treatment-naive patients (*n* = 6), platelet counts at the reference analysis were in the normal/mild range for two patients, whereas the remaining four patients had either moderate (*n* = 2) or severe (n = 2; platelet counts ≤ 60 × 10^9^/L) thrombocytopenia. Platelet counts had increased (Δ: +40.6 ± 26.1) for all the treatment-naive patients at the last analysis, with all patients having platelet counts within the normal range (mean count: 215.7 ± 126.8 × 10^9^/L).

As expected, the mean levels of chitotriosidase activity reported in the study population were much higher than those reported in individuals without GD in all analyses, but did decrease over the course of the study. Chitotriosidase activity levels decreased in both patient groups, with, as expected, a greater decrease among the treatment-naive patients (Δ −19,403.8 nmol/mL; slope: −2397.5 nmol/mL/h per year) than among the treatment-switched patients (Δ −968.8 nmol/mL; slope: −160.5 nmol/mL/h per year).

## 4. Discussion

This retrospective–prospective study using real-world data to assess the evolution of bone disease in patients with GD1 being treated with velaglucerase alfa provided valuable insights into the impact of the treatment, and into the quality and effectiveness of patient monitoring in clinical practice in France. Despite the publication of a detailed protocol for the monitoring of bone disease in patients with GD, we found that the quality of bone MRI data collected in clinical practice was often insufficient to allow for semiquantitative assessments of treatment responses through calculation of BMB scores. However, the centralized expert-radiologist qualitative assessment of real-world MRI data used in this study provided evidence that supports the results of clinical studies indicating the positive impact of velaglucerase alfa on bone disease, with improvements in bone infiltration being observed in treatment-naive patients and stabilization of bone infiltration being observed in treatment-switched patients. Furthermore, no new episodes of acute bone pain were reported. Improvements or stabilization of hematologic parameters and visceral manifestations were observed, providing further evidence from clinical practice of the effectiveness of velaglucerase alfa in allowing patients to achieve and maintain the well-established goals for GD treatment.

Assessing the extent of bone marrow infiltration in patients with GD is essential for evaluating the extent of bone involvement, monitoring patient responses to ERT, and for guiding therapeutic decision making and optimizing treatment regimens [3,39]. The BMB score, which provides an MRI-based semiquantitative evaluation of bone marrow infiltration based on the distribution of lesions and the change in signal intensity, is one of the most widely used methods in clinical studies [3,40]. This method has the advantage of being more reflective of whole-body bone marrow involvement because it includes assessment of the spine and the femur, and is simpler to use and more widely accessible than Dixon quantitative chemical-shift imaging (QCSI) assessments of the bone marrow fat fraction because it uses conventional MRI imaging [13,16,36,39,41]. However, the interobserver agreement of BMB scores has been questioned, even between experienced radiologists [42]. In addition, BMB scoring has been found to be less reliable for assessing bone infiltration in younger patients with GD, due to potential masking of the true extent of bone infiltration by the higher proportion of red bone marrow normally present in children and young adults [3,43,44]. The findings from the current study have highlighted the problems associated with the use of the BMB scoring for monitoring bone involvement in GD in real-world clinical practice. Longitudinal assessments of BMB scores rely on the collection of high-quality sequential MRI data according to a rather strict protocol, to ensure consistency between device settings and scanning parameters during follow-up. The MRIs used in this study were conducted by local radiologists using a range of MRI machine models from multiple centers and varied scanning parameters, resulting in large technical variations in the quality of the images obtained. Despite the availability of detailed procedures for conducting bone MRIs in French patients with GD [35], the centralized analysis of the MRI data revealed that in many cases these good practice guidelines were not followed, particularly the recommendations to collect image sequences of both the distal and proximal femur and to acquire T1- and T2-weighted sequences without fat-suppressed imaging to allow optimal comparison of the relative changes in signal intensity between healthy- and diseased-bone areas. These findings indicate that more specialized training needs to be provided to local radiologists on the acquisition and interpretation of data for calculating BMB scores. In countries where resources permit, this may need to be combined with centralized analysis through a national reference platform to limit interobserver variability in BMB scoring, or alternatively, the development of semi-automated techniques could be investigated to improve consistency.

As a result of the technical limitations associated with data acquisition, only one of the 17 adult patients included in the current study had interpretable data for femur BMB scoring, and only seven patients had interpretable data for spine BMB scoring. This absence of adequate data for the assessment of total BMB score led us to explore whether a subjective qualitive assessment of the change in bone infiltration (classified as worsened, improved or stabilized) would provide a more feasible method for analyzing the response to velaglucerase alfa treatment in clinical practice. The results of the centralized analysis indicated that this qualitative method could indeed be used to monitor changes in bone infiltration in the spine and femur over time, and to identify statistically significant differences between treatment groups. In addition, the centralized qualitative analysis by the expert radiologist also appeared to provide valuable information about the change in bone infiltration in the three children included in the study cohort. Although the normal developmental changes in red marrow made it more challenging to assess improvements in bone infiltration in these younger patients compared to those in the adults with GD, the masking effect of bone marrow maturation was not considered to have prevented the visualization of significant worsening of bone infiltration during the centralized expert-radiologist qualitative assessment. Thus, although larger validation studies are needed, our findings suggest that the qualitative bone infiltration analysis used in this study could provide an alternative, less-stringent, and easier-to-interpret method that could be used by local radiologists to assess bone disease in patients with GD, particularly in countries or regions where medical resources are scarce and access to GD expert radiologists is extremely limited.

The results of the centralized expert-radiologist qualitative analysis provided valuable real-world insights into the impact of velaglucerase alfa treatment on bone involvement in patients with GD, with treatment-naive patients and those with shorter treatment durations (3.5 years on average) tending to show improvements in femur and spine infiltration, and patients who switched treatments and treatment-naive patients with longer treatment durations (6 years on average) generally having stable bone disease. Importantly, none of the patients showed signs of worsening bone disease during velaglucerase alfa treatment. The results of this qualitative analysis are therefore consistent with findings of previous clinical studies in which assessment of BMB scores showed that velaglucerase alfa treatment led to a significant reduction in bone infiltration [13], with the largest reductions being observed during the first 5 years of ERT, followed by long-term stabilization after 5 years [15,16]. As pointed out in these studies [15,16], the stabilization of bone infiltration after 5 years of treatment suggests the need to revise current bone MRI monitoring protocols, perhaps increasing the interval between MRI evaluations for bone infiltration in patients who are adherent to treatment and have stable bone disease. Indeed, the current PNDS guidelines recommend bone MRI at treatment initiation, after 1 year, and then every 2 years once the disease has stabilized [26]. Improving adherence to the PNDS guidelines and ensuring that patients are monitored by MRI, particularly when initiating ERT or switching between therapies, is essential to allow for meaningful evaluation of treatment responses and for evidence-based updates of current monitoring recommendations.

In addition to improving bone infiltration, reducing bone pain is one of the key therapeutic goals in patients with GD [24,26]. Our analysis of clinical medical records indicated that this goal was met in our cohort: no new cases of acute bone pain were reported during velaglucerase alfa treatment, and the resolution of acute bone pain was reported in two patients. Chronic pain is also common in patients with GD, often associated with sequalae from previous bone events or in some cases linked to secondary events, such as secondary osteoarthritis, hip replacement, or the need to replace an existing prosthetic [3]. Although available data on pain severity were limited, the velaglucerase alfa treatment also seemed to lead to a reduction in or stabilization of chronic bone pain. Similar findings have been reported previously in several studies examining the impact of the alternative ERT, imiglucerase, on bone pain in patients with GD [8,12,45].

Based on the analysis of bone imaging records written by local radiologists, the large majority of patients in our cohort had existing bone lesions before the initiation of velaglucerase alfa therapy. As noted in previous studies, ERT cannot reverse all of the existing bone manifestations of GD, most notably avascular osteonecrosis and disease-related complications such as secondary osteoarthritis and fracture deformities [3], and thus the goal of therapy is to prevent bone infiltration and the occurrence of new lesions [24,26]. Velaglucerase alfa treatment appeared to allow all the patients included in our study to achieve this goal. However, the occurrence of bone lesions in patients receiving ERT has been reported in previous studies [46,47,48,49]. In particular, a study using whole-body MRI indicated that bone complications are more common in patients with a longer interval between GD diagnosis and initiation of ERT [43]. Avascular osteonecrosis has also been found to be more frequent in patients that initiated ERT more than 2 years after GD diagnosis [50]. Indeed, in our study, the mean time between diagnosis and initiation of any form of GD treatment was 2.6 years in treatment-naive patients and 9.9 years in patients switching treatments. Thus, the early detection of new lesions and surveillance of the severity of existing lesions remains essential for patients receiving ERT, not only for guiding the adjustment and optimization of velaglucerase alfa treatment, but also to allow timely intervention with supportive therapies and interventions to manage chronic pain such as prosthetic replacement to maintain or improve patient mobility and quality of life. In addition, other bone complications not specifically related to GD were reported in over 50% of the patients in our study. Clearly, there is a need to ensure that these nonspecific bone lesions are not overlooked during monitoring of the complex bone manifestations of GD and that correct diagnosis and appropriate management are provided.

Hepatomegaly and splenomegaly are hallmark manifestations of GD1, present in between 60% and 90% and more than 90% of cases, respectively [1]. The PNDS recommends regular monitoring of liver and spleen volumes every 6 months during the first year of treatment and then biannually after stabilization of organ volumes, by either abdominal MRI or ultrasound [26]. Although ultrasound has the advantage of being more accessible and affordable than other imaging modalities, it provides a less-comprehensive assessment of organ involvement than MRI [44]. In contrast, MRI data can be analyzed using semi-automated methods for measuring organ volumes, improving measurement accuracy and reproducibility [51]. In our study fewer than half of the patients had available abdominal MRI data collected around the time of initiation of velaglucerase alfa treatment and only around half of the patients had longitudinal abdominal MRI data. However, contrary to the bone MRIs, all the abdominal MRIs conducted provided interpretable, although not always optimal, cross-sectional data for the centralized analysis of organ volumes, demonstrating that the semi-automated method used in this study was sufficiently robust to overcome the variations in scanning parameters and sequence types associated with MRI data collected in clinical practice. Thus, while clinical examination and ultrasound may be sufficient for routine long-term management when MRI facilities are scarce, when MRI is widely available this technique could be used in clinical practice to monitor visceral disease severity and treatment responses.

The treatment goals for the visceral complications in GD1 are to reduce (within the first two years of treatment) and then stabilize organ volumes [26], ideally to within less than 1.0 to 1.5 MN for the liver and 2 to 8 MN for the spleen [24]. The centralized analysis of liver and spleen volumes demonstrated that these goals were met in our patient cohort, with treatment-naive patients showing decreases in liver and spleen volumes by the last visit to achieve average volumes of 1.2 MN and 3.9 MN, respectively, and treatment-switched patients showing stabilization of organ volumes (0.8 MN for the liver and 2.6 MN for the spleen at the last visit). These findings are therefore consistent with those of previous studies showing that velaglucerase alfa treatment leads to near-normalization of hepatomegaly and major reductions in splenomegaly within around 4 years of initiating treatment [11,52,53].

The same pattern of improvement in treatment-naive patients and stabilization in patients switching to velaglucerase alfa was observed for hemoglobin concentrations and platelet counts during the study. All patients had normalization or stabilization of hemoglobin and platelet counts at the last visit. Such normalization and maintenance of hematologic parameters in the 4 years after initiating treatment has been reported previously in patients receiving velaglucerase alfa [11], and has often been observed within the first 2 years post treatment initiation [52]. These findings are consistent with those of previous studies clearly demonstrating the effectiveness of velaglucerase alfa treatment in allowing patients to achieve the therapeutic goals of preventing anemia and reducing bleeding tendency, as well as the complications related to these hematologic manifestations [24,26,53]. Finally, monitoring of chitotriosidase activity, a well-established biomarker of GD severity and treatment responses [48,54,55], revealed decreases in activity in both the treatment-naive and treatment-switched patients.

The real-world ambispective design of the current study allowed the long-term impact of velaglucerase alfa treatment in GD1 to be assessed in clinical practice, in a patient population that was homogeneous in terms of the dose and frequency of velaglucerase alfa treatment received, and in both treatment-naive patients and those who had switched treatment. However, this real-world approach led to several study limitations. First, the number and quality of the MRIs conducted in clinical practice were insufficient to allow assessment of the primary study endpoint (i.e., the change in BMB scores). However, this lack of available data led us to explore the potential of an alternative and less-stringent qualitative method for assessing bone marrow involvement. Future studies would allow us to further evaluate the suitability of this method, or indeed of alternative approaches (e.g., the Spanish-MRI score [56]) for monitoring the bone marrow response to ERT in real-world clinical practice, and to more closely examine the suitability of the qualitative method for use in younger patients. Second, the size of the study population was small, and limited the power of the study to detect statistically significant between-visit differences and between-parameter correlations for some measures, most notably in the occurrence of bone pain, and to examine correlations between bone manifestations or genotypes and disease biomarkers, or evaluate the impact of recommended concomitant treatments on the study outcomes. The small size of the study population was associated with several factors. GD is a rare disease with an estimated prevalence of 1 in 140,000 in France [27]. According to the CETG registry, there were 97 patients with GD living in France who had received at least one dose of velaglucerase alfa at the time of the study. Among the centers managing these patients, only those treating more than one patient were invited to participate and only patients with GD1 and digital records of MRIs conducted within the 5 years preceding velaglucerase alfa initiation, or within the 3 months following treatment initiation, were eligible for study inclusion. Future studies involving larger populations would help to further clarify the extent to which ERT can prevent bone infiltration, and allow more detailed characterization of patients who are at highest risk of bone disease progression despite treatment. Ideally, a more organized, well-funded, international approach is required, perhaps using a purpose-designed platform for data collection. However, to allow pooling of all the collected data it is important that consensus is reached on MRI monitoring protocols and on the terminology used to describe the bone lesions, with the terms osteonecrosis, avascular necrosis, aseptic osteonecrosis and bone infarct often being used interchangeably in the literature, regardless of the anatomical location of the lesion. The size of the population also did not allow for a separate evaluation of disease evolution in patients that had undergone splenectomy, although the impact of this intervention on treatment responses has been reported previously [11]. Third, the study concentrated on data on bone involvement collected by MRI, as such other indicators of bone involvement (e.g., bone mineral density), were not analyzed as part of this study. Fourth, due to the retrospective nature of part of the study, the only GD marker with a sufficient amount of data available for longitudinal analysis was chitotriosidase, as more recently validated markers, such as glucosylsphingosine (lyso-Gb1) [57], were not commonly used in clinical practice at the time when many of the patients included in the study initiated velaglucerase alfa treatment. Fifth, the causes of chronic bone pain in GD are complex and the data on pain status evaluated in this study were subjective, based on records from clinical examinations performed as part of the regular monitoring of the patients rather than from study-specific pain assessments. Finally, the duration of the interval between the first and last available measure varied for each variable, reflecting the monitoring regimen of individual patients.

## 5. Conclusions

This study provided useful real-word data indicating the long-term effectiveness of velaglucerase alfa for the treatment of GD1 bone manifestations in treatment-naive and -switched patients. Our findings also highlighted the difficulties associated with using BMB scores for monitoring bone treatment responses in routine clinical practice. Specialized training for local radiologists and/or a centralized analysis reference platform may improve bone monitoring. The simplified qualitative bone-infiltration analysis method used in the current study indicated that bone infiltration improved in treatment-naive patients and remained stable for treatment-switched patients. In addition, improvements in bone infiltration were observed in patients with shorter treatment durations (<3.5 years with velaglucerase alfa alone or velaglucerase alfa plus a prior GD treatment), whereas for patients with longer treatment durations (>6 years) bone infiltration remained stable. The results of this centralized expert-radiologist qualitative analysis therefore support those of previous clinical studies suggesting that the interval between MRI evaluations can be increased in patients with long-term stabilization of bone infiltration. Although larger validation studies are needed, our findings suggest that when a GD expert radiologist is not available, an easier qualitative analysis method could be a valuable tool for routine bone monitoring in clinical practice. Evaluation of the clinical characteristics of the patients suggested that velaglucerase alfa treatment also appeared to be associated with a reduction in bone pain. Similar patterns of improvement and stabilization were observed in the two groups for the reduction in liver and spleen volumes and hematologic parameters.

## Figures and Tables

**Table 1:** Demographic, clinical and MRI characteristics of the patients.

Patient Demographics and Disease History at Inclusion	*n* (%) or Mean ± SD (Min–Max)
Age (years)	46.0 ± 18.7 (8.0–75.0)
Gender (M/F)	11 (55)/9 (45)
Age of diagnosis of Gaucher disease (years)	22.3 ± 15.3 (1.0–52.0)
Family history of Gaucher disease (Y/N)	7 (35.0)/13 (65.0)
Splenectomy (Y/N)	3 (15)/17 (85)
**Predicted pathogenic GBA variants, *n* = 16:**	***n* (%)**
homozygous p.Asn409Ser	1 (6.3))
p.Asn409Ser/ND	3 (18.8).
p.Asn409Ser/p.Leu483Pro	2 (12.5)
p.Asn409Ser/RecNcil	2 (12.5)
p.Asn409Ser/other ^1^	4 (25.0)
homozygous p.Leu483Pro	1 (6.3)
IVS2 + 1G > A/p.Asn409Ser	1 (6.3)
p.Ala127Val/RecNcil	1 (6.3)
**Treatment patterns**	***n* (%) or Mean ± SD (Min–Max)**
Time between diagnosis and velaglucerase alfa initiation (years), *n* = 20	16.6 ± 14.6 (0.0–59.0)
Use of treatment for Gaucher disease prior to velaglucerase alfa (Y/N)	14 (70)/6 (30)
Duration of use of prior treatment for Gaucher disease (years), *n* = 14	11.3 ± 5.5 (1.2–19.3)
Treatment before switching to velaglucerase alfa:	
Imiglucerase	13
Miglustat	1
**Clinical characteristics at velaglucerase alfa initiation**	***n* (%) or Mean ± SD (Min–Max)**
BMI (kg/m²), *n* = 14	20.7 ± 4.1 (14.2–27.1)
Chronic pain in bone (Y/N)	6 (37.5)/10 (62.5)
Acute bone pain (Y/N)	2 (15.4)/11 (84.6)
Clinical signs of hepatomegaly (Y/N)	3 (50.0)/3 (50.0)
Clinical signs of splenomegaly (Y/N)	5 (62.5)/3 (37.5)
**Biological analyses at velaglucerase alfa initiation**	***n* (%) or Mean ± SD (Min–Max)**
Hemoglobin concentration (g/dL), *n* = 16	13.3 ± 2.5 (7.9–17.1)
Platelets (×10^9^/L), *n* = 16	133.6 ± 91.9 (26.0–356.0)
Ferritin (μg/L), *n* = 10	480.2 ± 579.4 (77.0–1894.0)
Chitotriosidase activity (nmol/mL/h), *n* = 10	8663.3 ± 14370 (968.0–48750.0)
Monoclonal gammopathy (Y/N)	2 (11.8) /15 (88.2)
**Disease imaging characteristics at the time of the reference MRI ^2^**	***n* (%) or Mean ± SD (Min–Max)**
Presence of bone lesions (Y/N) ^3^	17 (89.5)/2 (10.5)
Hepatomegaly (Y/N)	7 (70)/3 (30)
Estimated liver volume (mL), *n* = 7	1691.2 ± 809.2
Estimated liver volume, multiples of normal ^4^	1.3 ± 3.8 (0.7–3.0)
Splenomegaly (Y/N)	10 (83.3)/2 (16.7)
Estimated spleen volume (mL), *n* = 7	843.0 ± 1206.3
Estimated spleen volume, multiples of normal ^4^	9.9 ± 53.1 (1.2–38.7)

^1^ Among c.1226A > G (p.Asn409Ser) heterozygous carriers, other alleles included the following predicted variants (each found in one patient): p.Arg535Pro, p.Leu29AlafsX18, a complex mutant allele (p.Asn227Arg, p.Val230Gly, p.Ser235Pro, and p.Gly241Arg), p.Leu483Arg, and p.Gly421ThrfsX28. ^2^ The reference MRI was the MRI performed closest to the time of velaglucerase alpha (conducted within the 5 years preceding velaglucerase alfa initiation, or within the 3 months following velaglucerase initiation). ^3^ Bone lesions were reported based on records made by local radiologists and included Erlenmeyer flask-shaped deformation, bone infiltration, cortical thinning, lytic lesions, avascular osteonecrosis (defined as an infarct located in epiphysis) and bone infarct (defined as an infarct located in long bone: metaphysis or diaphysis, or flat bone), vertebral collapse, fracture, secondary osteoarthritis, and osteosynthesis. ^4^ For multiples of normal, the normal liver volume was 25 mL/kg of body weight, and the normal spleen volume was 2 mL/kg of body weight. Abbreviations: BMI, body mass index; F, female; M, male; min–max: minimum and maximum values; n, number of patients with available data; ND; not determined; SD, standard deviation.

**Table 2:** Qualitative change in spine and femur infiltration between the first and last available MRIs according to the time between MRIs, duration of velaglucerase alfa treatment, and the duration of all treatments for Gaucher disease (velaglucerase alfa and any prior treatment).

Patients with MRI Data (*n* = 19) ^1^	Time (Years ± SD)		
Spine Infiltration	Improved *n* = 4	Stable *n* = 14	Worsened *n* = 0
Time between first and last MRI	3.7 ± 0.7	5.2 ± 3.0	0
Treatment duration:			
Velaglucerase alfa alone	3.5 ± 0.3	6.0 ± 2.5	0
Velaglucerase alfa plus prior GD treatment	3.5 ± 0.3	17.0 ± 7.2	0
Femur Infiltration	Improved *n* = 5	Stable *n* = 13	Worsened *n* = 0
Time between first and last MRI	6.3 ± 3.7	4.5 ± 2.6	0
Treatment duration:			
Velaglucerase alfa alone	4.7 ± 1.7	5.7 ± 2.6	0
Velaglucerase alfa plus prior GD treatment	10.1 ± 9.2	15.5 ± 8.2	0

^1^ MRI data were uninterpretable for the centralized expert-radiologist qualitative analysis for one patient. Abbreviations: GD, Gaucher disease; SD, standard deviation.

**Table 3:** Review of bone imaging and clinical characteristics from local imaging and clinical records between the reference visit and the last available follow-up visit.

**Bone Imaging Characteristics ^1^ *n* = 19**	**Reference Visit ^4^** ***n* (%)**	**Last Available Visit** ***n* (%)**	***p*** **Value ^6^** **Change Reference to Last**	**Change Reference to Last (*n* ↑↓) ^7^**	
				**Treatment-Naive Patients (** ***n* = 6)**	**Treatment-Switched Patients** **(** ***n* = 14)**
Presence of bone lesions ^2^ (Y/N)	17 (89.5)/2 (10.5)	17 (89.5)/2 (10.5)	N/A	↔	↔
Bone infiltration (Y/N)	11 (57.9)/8 (42.1)	11 (57.9)/8 (42.1)	1.0	↑1 ^8^ ↓1	↔
**Clinical Bone Characteristics ^3^**	**Reference Visit ^5^** ***n* (%)**	**Last Available Visit** ***n* (%)**	***p*** **Value ^6^** **Change Reference to Last**	**Treatment-Naive Patients (*n* = 6)**	**Treatment-Switched Patients (*n* = 14)**
Chronic bone pain (Y/N), *n* = 16	8 (50)/8 (50)	6 (37.5)/10 (62.5)	0.5	↑1 ↓1	↑2 ↓4
Acute bone pain (Y/N), *n* = 4	2 (14.3)/12 (85.7)	0 (0)/14 (100)	N/A	↑0 ↓1	↑0 ↓1

^1^ Average time between the reference visit and last visit for bone imaging: 6.6 years ± 3.4 years. ^2^ Bone lesions were reported from records made by local radiologists and included Erlenmeyer flask-shaped deformation, bone infiltration, cortical thinning, lytic lesions, avascular osteonecrosis (defined as an infarct located in epiphysis) and bone infarct (defined as an infarct located in long bone: metaphysis or diaphysis, or flat bone), vertebral collapse, fracture, secondary osteoarthritis, and osteosynthesis. ^3^ Average time between the reference clinical examination and last clinical examination: 6.7 years ± 3.4 for chronic bone pain and 5.9 years ± 3.2 for acute bone pain. ^4^ For imaging analyses, the reference visit was either the reference MRI (the MRI conducted closest to velaglucerase alfa initiation and within 5 years before or 3 months after velaglucerase alfa initiation) or other bone imaging modality conducted within 3 months of the reference MRI. ^5^ For the clinical analyses, the reference visit was the clinical examination performed within 3 months of the reference MRI. ^6^ *p* values were calculated using the McNemar chi-square test. ^7^ ↑ number of patients with a change from the reported absence of this lesion at the reference visit to the reported presence of this lesion at the last visit. ↓ number of patients with a change from the reported presence of the lesion at the reference visit to the reported absence of this lesion at the last visit. ↔ no change for all patients. ^8^ For the patient in which bone infiltration was reported to be absent at the first MRI but present at the last MRI by local radiologists, the MRIs were deemed uninterpretable by the centralized expert-radiologist qualitative analysis. Abbreviations: n, number of patients with data available for both visits; N/A, *p* value not available.

**Table 4:** Change in liver and spleen parameters over the course of the study.

Hepatosplenomegaly ^1,2,3^		Reference Visit *n* (%)	Last Visit *n* (%)	Change between Reference and Last Visit *n* (% Total Y/N) ^5^	*p* Value ^6^
Hepatomegaly on clinical examination (Y/N), *n* = 12		4 (33.3)/8 (66.7)	3 (25.0)/9 (75.0)	**↑** 1 (33.3) **↓** 2 (22.2)	0.2
Hepatomegaly by MRI (Y/N), *n* = 7		4 (57.1)/3 (42.9)	3 (42.9)/4 (57.1)	**↑** 0 (0) **↓** 1 (25.0)	0.3
Splenomegaly on clinical examination (Y/N), *n* = 11		6 (54.5)/5 (45.5)	1 (9.1)/10 (90.9)	**↑** 1 (100.0) ↓ 6 (60.0)	0.5
Splenomegaly by MRI (Y/N), *n* = 9		7 (77.8)/2 (22.2)	5 (55.6)/4 (44.4)	**↑** 0 (0.0)/↓ 2 (50.0)	0.2
**Estimated Liver Volume, Centralized Analysis ^4^**		**First MRI** **Mean ± SD**	**Last MRI** **Mean ± SD**	**Change First and Last MRI** **Mean ± SD**	***p*** **Value ^7^**
Whole population, *n* = 12	mL	1617.8 ± 644.8	1523.4 ± 376.2	−94.4 ± 440.6	0.5
	MN	1.09 ± 0.63	0.93 ± 0.23	0.17 ± 0.5	
Treatment-naive patients, *n* = 3	mL	2061.1 ± 1166.2	1498.5 ± 395	−562.6 ± 777.0	0.1
	MN	1.8 ± 1.1	1.2 ± 0.23	−0.6 ± 0.8	
Treatment-switched patients, *n* = 9	mL	1470.0 ± 365.2	1531.7 ± 394.0	61.7 ± 79.7	
	MN	0.9 ± 0.2	0.8 ± 0.1	0.1 ± 0.1	
**Estimated Spleen Volume, Centralized Analysis ^4^**		**First MRI** **Mean ± SD**	**Last MRI** **Mean ± SD**	**Change First and Last MRI** **Mean ± SD**	***p*** **Value ^7^**
Whole population, *n* = 10	mL	776.6 ± 992.7	385.9 ± 199.6	−390.7 ± 863.9	0.001
	MN	16.3 ± 19.8	3.9 ± 2.8	−12.4 ± 17.4	
Treatment-naive patients, *n* = 3	mL	1497.7 ± 1764.6	398.1 ± 296.1	−1099.6 ± 1497.4	0.3
	MN	16.3 ± 19.8	3.9 ± 2.8	−12.4 ± 17.4	
Treatment-switched patients, *n* = 7	mL	467.5 ± 262.4	380.7 ± 174.5	−86.9 ± 114.6	
	MN	3.2 ± 1.4	2.6 ± 1.0	−0.7 ± 0.7	

^1^ Three patients had undergone splenectomy. ^2^ Mean time between the reference clinical examination and the last available clinical examination: 5.8 years ± 3.9 for hepatomegaly and 6.2 ± 3.8 for splenomegaly. ^3^ Mean time between the reference MRI and the last available MRI: 5.9 ± 4.2 for hepatomegaly and 6.6 ± 4.1 for splenomegaly. ^4^ Mean time between the first available MRI and the last available MRI for volume estimates: 5.6 years ± 2.9 for liver measurements and 5.6 years ± 3.1 for spleen measurements. ^5^ ↑ change from the reported absence of this manifestation at the reference visit to the reported presence of this manifestation at the last visit. ↓ change from the reported presence of this manifestation at the reference visit to the reported absence of this manifestation at the last visit. ^6^ *p* values calculated using the McNemar chi-square test. ^7^ *p* values calculated using the Wilcoxon rank test. Abbreviations: MN, multiples of normal (25 mL/kg of body weight for the liver 2 mL/kg of body weight for the spleen); *n*, number of patients with data available for both visits; SD, standard deviation.

**Table 5:** Change in biological parameters over the course of the study.

Parameter	Reference Analysis Mean ± SD	Last Analysis Mean ± SD	Change between Reference and Last Mean ± SD	Slope Units/Year	*p* Value (Δ Ref to Last) ^3^
Hemoglobin concentration (g/dL) ^1^					
All patients, *n* = 19	13.2 ± 2.2	13.8 ± 1.6	0.6 ± 1.5	0.3 ± 0.9	0.08
Treatment-naive patients, *n* = 6	10.8 ± 1.7	12.8 ±1.0	2.0 ± 1.3	1.1 ± 1.3	--
Treatment-switched patients, *n* = 13	14.3 ± 1.3	14.3 ± 1.6	0 ± 1.2	−0.1 ± 0.4	
*p* value (naive—non-naive patients at each analysis) ^2^	<0.0001	0.05	0.005	0.007	--
Platelet counts (×10^9^/L) ^1^					
All patients, *n* = 18	139.1 ± 76.5	185.2 ± 77.6	46.1 ± 42.7	14.1 ± 25.3	0.0003
Treatment-naive patients, *n* = 6	123.8 ± 119.3	215.7 ± 126.8	91.8 ± 28.2	40.6 ± 26.1	--
Treatment-switched patients, *n* = 12	146.8 ± 48.8	169.9 ± 35.2	23.2 ± 27.3	0.76 ± 10.2	
*p* value (naive—treatment-switched patients) ^2^	0.6	0.3	0.0001	0.0002	--
Chitotriosidase activity (nmol/mL/h) ^1^					
All patients, *n* = 13	9084.0 ± 16,039.5	2442.9 ± 5031.8	−6641.1± 16,831.2	−2.3 ± 16.6	0.02
Treatment-naive patients, *n* = 4	25,688.3 ± 22,224.4	6284.5 ± 8408.2	−19,403.8 ± 28,604.8	−2397.5 ± 11,939.5	--
Treatment-switched patients, *n* = 9	1704.3 ± 1248.7	735.6 ± 898.9	−968.8 ± 677.4	−160.5 ± 138.7	
*p* value (naive—treatment-switched patients) ^2^	0.007	0.14	0.19	0.19	--

^1^ Mean time between the reference biological analysis and the last available biological analysis: 6.6 ± 3.6 for measurement of hemoglobin, 6.1 ± 3.4 years for platelet counts, 5.9 ± 3.4 for chitotriosidase activity. ^2^ *p* values for the difference between values obtained for treatment-naive and treatment-switched patients at each analysis and for the slope were obtained using the *t* test for hemoglobin concentration and platelet counts, and the Wilcoxon rank test for chitotriosidase activity. ^3^ *p* values for the difference in values at the reference and last analysis obtained using the t test for hemoglobin concentration and platelet counts and the Wilcoxon rank test for chitotriosidase activity. Abbreviations: *n*, number of patients with data available for both visits; Ref, reference analysis; SD, standard deviation.

## Data Availability

The datasets used and/or analyzed during the current study are available from the corresponding author on reasonable request.
